# Alleviating biotic stress of powdery mildew in mango cv. Keitt by Sulfur nanoparticles and assessing their effect on productivity and disease severity

**DOI:** 10.1038/s41598-025-88282-z

**Published:** 2025-02-14

**Authors:** Mohamed K. Abou El-Nasr, Mohamed A. Nasser, Mohamed Ebrahim, Mina S. F. Samaan

**Affiliations:** 1 Department of Horticulture, Faculty of Agriculture, Ain Shams University, 68-Hadayek Shoubra, Cairo, 11241 Egypt; 2 Department of Plant Pathology, Faculty of Agriculture, Ain Shams University, 68-Hadayek Shoubra, Cairo, 11241 Egypt

**Keywords:** Keitt, Mango, NPs, Sulfur, Nanoparticles, Biotic stress, Plant sciences, Plant physiology, Plant stress responses

## Abstract

The control of powdery mildew disease is one of the main objectives in Mango production. Mango production with superior quality is becoming increasingly challenging due to climate change, which may negatively affect all stages of their development. Recently, Nanotechnology is a promising and rapidly evolving field that could be a very useful tool to raise the efficiency of fungicides. The aim of this study was to evaluate the role of sulfur nanoparticles (SNPs) and reducing the incidence of powdery mildew, improving growth performance and productivity of mango cv. Keitt (*Mangifera indica* L.). Four concentrations of SNPs (0, 100, 300, and 500 ppm) were compared to sulfur microform (bulk) at a concentration of 500 ppm. At a private farm on Cairo Alex Desert Road K78, Egypt, foliar spray treatments were administered to an eight-year-old mango cv. Keitt grafted on Sukari rootstock. The experiment was conducted using a randomized complete block design, with each tree receiving a five-liter spray of the solution. The results suggested that the use of 500 ppm concentration of SNPs led to reducing powdery mildew disease incidence by 8.8% but it negatively affected productivity, as it decreased by 27% compared to the control treatment. Whereas 100 ppm concentration of SNPs led to increasing the productivity by 342% compared to the control treatment, reducing powdery mildew disease incidence in mango was recorded at 14.6%. Furthermore, the TSS and vitamin C increased significantly by 16 Brix and 46.3 mg, respectively, in association with decreasing titratable acidity in fruits. In aggregate, our data demonstrated that 100 ppm and 300 ppm of sulfur nanoparticles was more efficient than 500 ppm of sulfur bulk in improving POD and PPO enzyme activity. It is recommended to add SNPs of 100 ppm as foliar spraying three times at 15-day intervals for alleviating the harmful impact of disease on mango trees by improving the enzyme activity, thereby yield, and fruit quality of mango trees exposed to powdery mildew disease.

## Introduction

Mango (*Mangifera indica* L.) is an evergreen fruit tree belonging to the Anacardiaceae family, it’s native to Southeast Asia and is extensively planted in different climatic tropical or subtropical regions^1–3^. In Egypt, mango production in 2023 was estimated to be over 1.4 million metric tons from an area of around 125 thousand hectares, with an average fruit yield of about 11.4 ton per hectare^4^. Mango production in some world regions is facing challenges due to climate changes, such as high temperature, soil or irrigation water salinity that impacts on fruit quality and productivity^5,6^. With climate change, environmental conditions of temperature and humidity are becoming favorable for powdery mildew to occur, which in turn affects the amount of flowering and thus reduces productivity and yield. Mango powdery mildew is one cause of the widespread problem of poor mango fruit set and yield^7^.

Powdery mildew is a prevalent fungal disease that impacts numerous plant species, including mango trees. It’s caused by *Pseudoidium anacardii*^8,9^ formerly *Oidium mangiferae* Berthet, in mango trees^10–12^. It’s a major disease characterized by a powdery white fungal growth that covers plant leaves, inflorescence clusters, and young fruits, but the flowering stage appears to be the most susceptible to infection or during fruit set^8,13^ which can hinder photosynthesis and lead to reduced crop yields. Although the fungus is a biotrophic microorganism that does not directly cause plant cell death, as it depends on living cells to acquire nutrients and continue its life cycle^14^, through penetrating the plant cuticle and cell wall^15^. It may cause serious losses that could be up to 90% when blossoming and growth initiation that negatively affects productivity^16,17^. The pathogen develops in dry and cold conditions whilst becoming more severe at 90% relative humidity (RH) and temperature ranging between 20 and 25 °C^18,19^.

Disease control is mainly depending on using diverse fungicidal chemicals, but there is a risk that excessive use has detrimental impacts or reinforcing a resistant fungal by additional applications of ineffective fungicides^20^ on mango orchards in Egypt. Fungicides such as carbendazim, thiabendazole, and benomyl to which *P. anacardii* had evolved resistance^18,19,21^. However, fungicide rotation with different sites of action or multisite fungicides such as sulfur, dithiocarbamates, and quinomethionates can act efficiently and reduce the risk of the appearance of fungal resistance^18^. So, demand for alternative disease management strategies is heightened by the desire to reduce pesticide levels on food crops and environmental health concerns.

Sulfur may act as a phytoalexin and is considered one of the earliest fungicides; it has been used to resist powdery mildew since the nineteenth century^22,23^. It’s applied sulfur-based fungicides at the early stages of infection or as a preventive measure to achieve optimal results. By interfering with the enzymatic processes within the fungal cells, disrupting their ability to thrive and spread, sulfur can kill spores and mycelia; therefore, it’s useful as a therapeutic or preventive fungicide^24,25^. Sulfur penetrates the fungal cells, spores and into cytoplasm, damaging their structure and inhibiting the electron transport chain for respiration directly by modifying crucial protein thiols^26^. Sulfur is well recognized for its function in the synthesis of chlorophyll, proteins, and the amino acids as methionine and cysteine^27^. A concept was proposed to improve control of powdery mildews by using lower or safe doses of fungicides to reduce the risk of phytotoxicity, harm to non-target species, and environmental hazards^28,29^. There is a growing interest in developing nanomaterials for agricultural use, including new crop management techniques and the delivery of nutrients and pesticides^30,31^. The featured physicochemical characteristics of nanotechnology that act an effective improvement in the agricultural sector, where nanomaterials are being employed more frequently in agriculture to increase plant biomass owing to their tiny size and vast surface area^32^ or pesticides have been able to draw much attention due to their higher efficacy even at very low doses^28^. Notably, this nanomaterial has only recently been used on fruit crops, while several studies focused on field crops and a few vegetable crops^32^. Sulfur Nanoparticles (SNPs) have been utilized as fungicide and pesticide to combat some plant diseases but till date there are a few reports about that^33,34^. Besides, SNPs enhance the growth characteristics of some plants including Tomato^35,36^; Rapeseed^34^.

Our study hypothesis was that improving productivity and fruit quality of mango, powdery mildew disease must be suppressed as one of the production determinants in mango. Bearing the effectiveness of micronized sulfur as a preventive spray for the disease, nanometric sulfur can be more effective in suppressing the disease. Considering the above, the objectives of this study are to offer relevant data about the influence of SNPs spraying on morpho-physiological attributes, disease severity index, disease incidence percentage and its direct impact on the productivity and fruit quality.

## Materials and methods

### Preparation of sulfur nanoparticles (SNPs)

As per^37^, a co-precipitation approach was utilized to synthesize SNPs. Briefly: To prepare the SNPs, a sodium thiosulphate anhydrous (Na_2_S_2_O_3_) solution was mixed with hydrochloric acid (HCl) at room temperature (25 °C). The preparation of this solution involved dissolving 1.581 g of Na_2_S_2_O_3_ in 900 ml of distilled water and stirring the liquid until it turned transparent. 0.2 M HCl (17 ml/L) was added to the solution during stirring. After mixing the reactants, the solution was kept for 40 min in an ultrasonic bath for the completion of the reaction. The yellow precipitate was collected, filtrated by paper filter, and washed with distilled water several times, and then the precipitate was dried at 60 °C for 12 h.

### Characterization of sulfur nanoparticles (SNPs)

A scanning electron microscope (Quattro S, Thermo Scientific, Waltham, MA, USA) was utilized to analyze the surface morphology of SNPs. 10 mg of SNPs were suspended in 10 mL of deionized water and transferred to a 2 mL cuvette for measuring the average particle size distribution and potential (surface charge) by dynamic light scattering (DLS) by using the Zetasizer device (Malvern Pan Analytical, Westborough, MA, USA). The crystallographic phase pattern was determined using an X-ray diffractometer (XRD, X’Pert PRO Malvern-PANalytical, Etten Leur, Netherlands), and data analysis was carried out with a high score plus software^32^.

### Plant material and experimental design

The current study was carried out in a private orchard situated in Cairo Alex Desert Road K78, Egypt (30°17′27.0"N 30°32′21.3"E, altitude 56 m above sea level), during the 2023 season. Ten-year-old Keitt mango (*Mangifera indica* L.) cultivar grafted on Sukary rootstock and cultivated in sandy soil at a distance of 2 × 4 m under a drip irrigation system. SNPs were used at different concentrations (100, 300, and 500 ppm) compared to micronized sulfur concentration of 500 ppm. The control treatment involved spraying only with well water. The trees that were treated received three rounds of spraying, 15- to 30-day intervals among them at pre-bloom and full-bloom stages. Each tree received about 5 L of spraying solution until runoff. Branches with consistent flowering stages and bunch sizes were identified following sulfur application. The study was carried out using a randomized complete block design, with ten replications, and each replicate was exemplified by one tree. Fruits were harvested from the labeled branches at commercial maturity.

### Disease assessment

####  Disease incidence rate

The disease incidence rate was calculated as a number of infected inflorescences per tree according to^13^, where the percentage of disease incidence is calculated using the equation below:

$$\text{Disease incidence (\%)}= \frac{\text{No. of infected inflorescences}/\text{tree}}{\text{Total no. of inflorescenecs}/\text{tree}}\times100$$

#### Disease severity

**Fig. 1:**
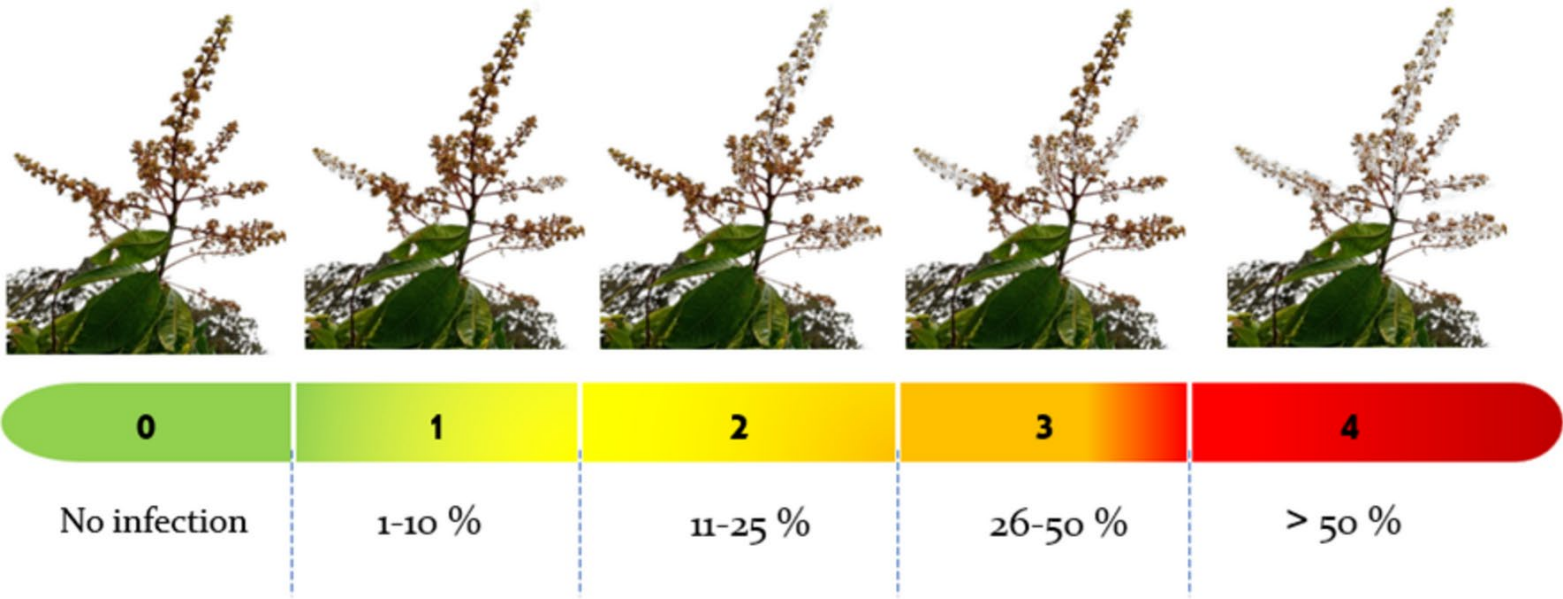
The powdery mildew scale (0–4) is designed for recording disease severity in mango panicles. 0 = no infections; 1 = 1–10%; 2 = 11–25%; 3 = 26–50%; 4 =  > 50%.

The disease severity index was rated on a scale for powdery mildew on inflorescence according to^10^, the percentage of area covered by powdery mildew colonies was divided into a five-category scale as shown in Fig. 1. Readings were recorded after 0, 14, and 28 days from spraying (m1, m2, and m3).

$$\text{Diseases severity}= \frac{\text{Sum of all the score of individual inflorescence}}{\text{Total no. of inflorescenec observed}}\times\frac{100}{\text{Maximum scale}}$$

Area under disease progress curve (AUDPC) was computed utilizing the below formula according to^38^.

$$AK= \sum_{i=1}^{Ni-1}\frac{(y{\text{i}}+y\text{i}+1)}{2}(t\text{i}+1-t{\text{i}})$$

### Determination of Sulphur (S) content of leaves

Samples of plants were dried for 24 h at 70 °C after being washed with distilled water. The process of grinding was initiated after drying. The ground samples were burnt in a combination of H_2_O_2_-HNO_3_ acid in the microwave oven (Milestone) for thirty minutes and filtered through a blue-banded filter paper. After the filtered samples had been diluted to a final volume of 20 ml with distilled water, S analysis was performed in Inductively Coupled Plasma (ICP) at a wavelength of 182.037 nm^39^.

### Tree productivity

At harvest stage, the total yield per tree was calculated by counting and weighing the fruits on each tree.

$$\text{Total yield per tree}=\text{number of fruits per tree}*\text{average of fruit weight}$$

### Fruit quality

Ten fruits were randomly selected to determine fruit length, fruit diameter, fruit shape, pulp weight, and peel thickness. In biochemical analysis of fruits, total soluble solids (TSS) was determined by utilizing a hand refractometer (HR-110) and titratable acidity (TA) was recorded as citric and malic acid as major organic acids in Keitt mango by 5 ml juice used in titration according to^40^, the maturity index (TSS:TA ratio) was calculated. Ten grams of pulp were mixed with 3% oxalic acid, and an aliquot was titrated against 2,6-dichlorophenolindophenol to determine the amount of vitamin C present. Vitamin C contents were expressed as mg/100 ml^41^.

### Oxidative stress markers

#### Polyphenol oxidase activity (PPO)

Polyphenol oxidase enzyme activity (IU/mL enzyme) was calculated by tracking the increase in the absorbance reading at the wavelength of 420 nm due to the formation of the benzoquinone compound as described by^42^. A leaf sample of 1 g was briefly ground in 4 mL of an extraction buffer composed of 0.1 M phosphate buffer, pH 7, with 0.1 mM EDTA and 1% polyvinylpyrrolidone (PVP). The extract was centrifuged for fifteen minutes under cooling. Enzyme activity was evaluated using the supernatant collected. To measure the enzyme activity, 2.3 mL of phosphate buffer was taken (pH 6.5, 0.1 M) solution, then 0.6 mL of 0.1 M catechol was added. Finally, 0.1 mL of the enzymatic extract was added. A change in absorbance of 0.001 per minute per millilitre of enzyme extract was used to define one unit of enzyme activity.

#### Peroxidase activity (POD)

The assay of peroxidase activity was conducted based on its capacity to transform guaiacol into tetra guaiacol according to^43^. In brief, a leaf sample of 1 g was ground in a 4 mL extraction phosphate buffer. The reaction mixture contained 2.9 mL of 100 mM phosphate buffer (pH 7.0), 20.1 mM guaiacol, 10 mM H_2_O_2_ and the enzyme extract was 0.1 mL. The increase in absorbance was recorded by the addition of H_2_O_2_ at 470 nm for 3 min.

#### H_2_O_2_ concentration

With minor adjustments, the concentration of hydrogen peroxide (H_2_O_2_) was measured in accordance with^44^. Three millilitres (mL) of 1% (w/v) tri-chloroacetic acid (TCA) was used to homogenize 0.5 g of fruit pulp samples. For ten minutes, the homogenate was centrifuged at 10,000 rpm and 4 °C. Then, 0.75 mL of the supernatant was combined with 1.5 mL of 1 M KI and 0.75 mL of 10 mM K-phosphate buffer (pH 7.0). The absorbance at 390 nm of H_2_O_2_ was measured and compared to a standard calibration curve. Using a standard curve that was plotted between 0 and 15 nmol mL^−1^, the concentration of H_2_O_2_ was determined.

### Statistical analysis

All the statistical analysis of the different traits were performed using the analysis of variance (ANOVA) method. Mean comparisons were carried out by Tukey test^45^ multiple range tests at *p* ≤ 0.05. The data were statistically analyzed using the analysis of variance with the SAS package. Using XLSTAT (version 2018.1), a biplot of principal component analysis (PCA) was generated to examine the relationships between the attributes studied.

## Results

### Characterization of sulfur nanoparticles (SNPs)

**Fig. 2:**
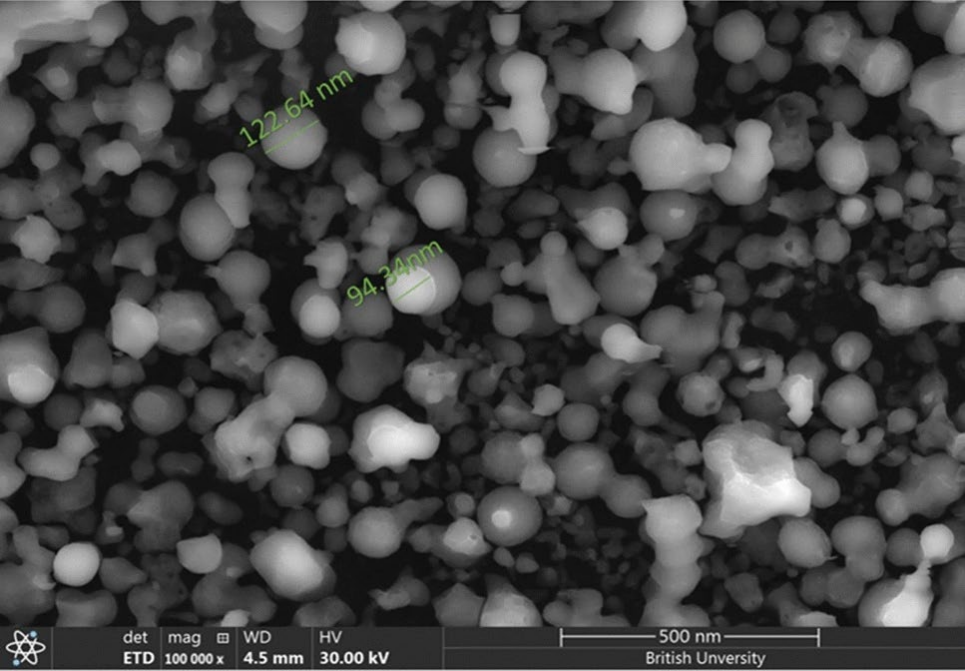
SEM image of the prepared S Nanoparticles shows that particles have spherical shape.

**Fig. 3:**
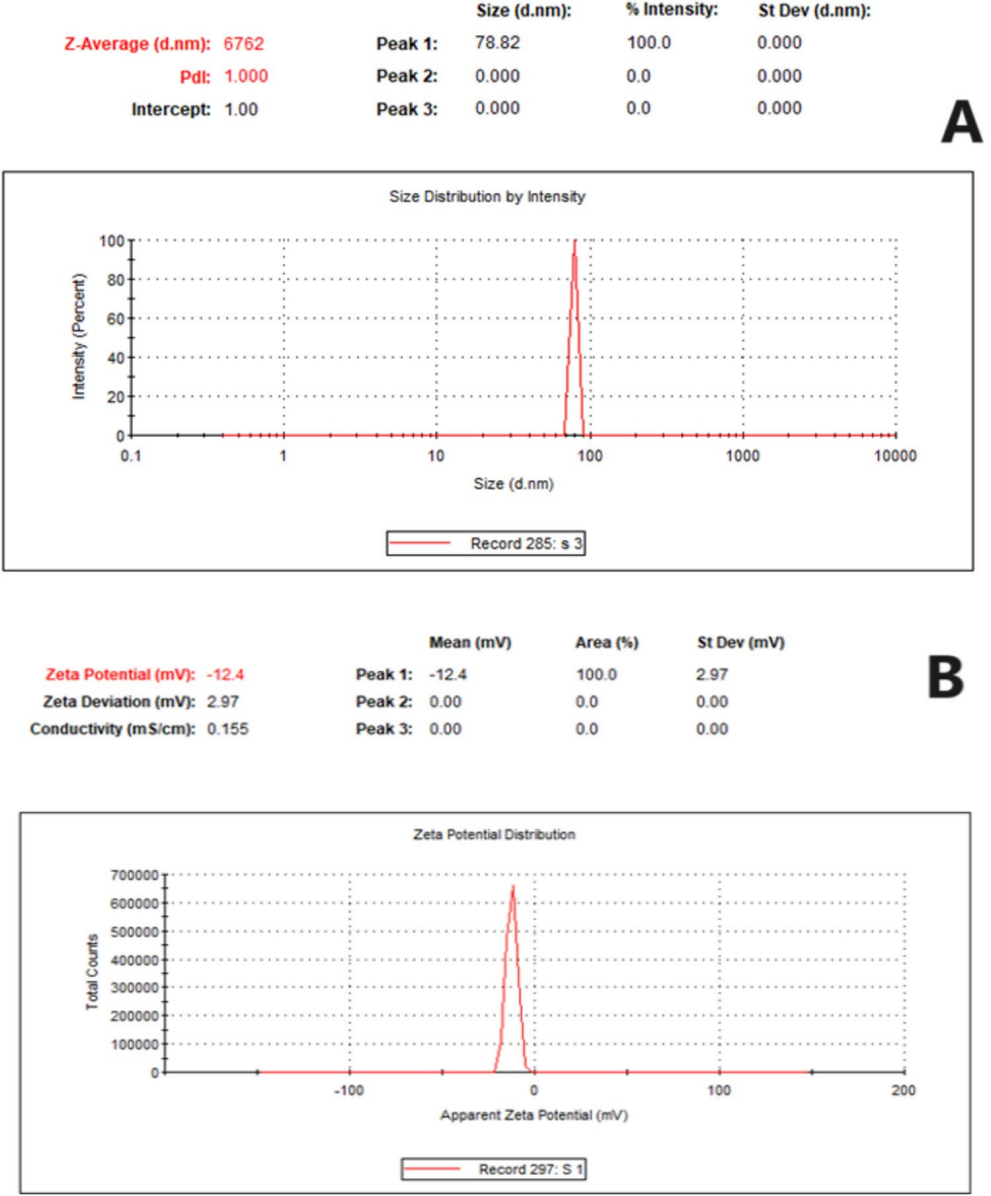
Physicochemical characterization of S nanoparticles. (**A**) Particle size distribution. (**B**) Zeta potential.

**Fig. 4:**
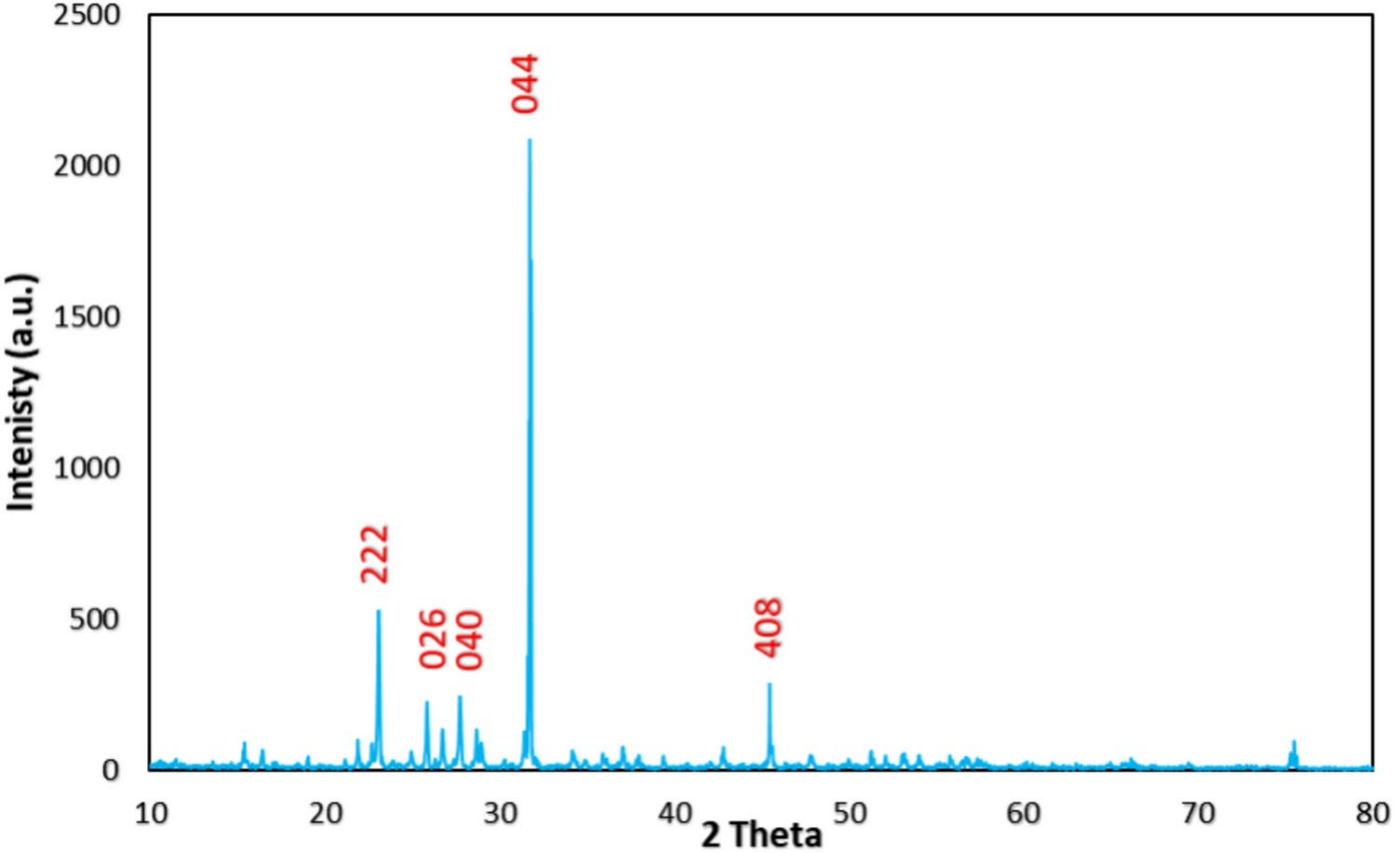
Graph represents the XRD pattern of SNPs crystal.

Sulfur nanoparticles were synthesized via a co-precipitation method at an acidic pH with the use of hydrochloric acid as a stabilizing and reducing agent as previously described. The shape morphology of SNPs was examined via topographical SEM image, and the SNPs were found to be sphere shaped with a size of 85 nm (Fig. 2). As shown in Fig. 3A and B, a ζ-potential of − 12.4 mV and an average particle size of 78.82 nm were obtained via dynamic light scattering (DLS). Additionally, the crystal size was determined as shown in Fig. 4. Where, XRD patterns were compared to the standard pattern of S (card #: 00-008-0247), which showed that the diffraction peaks at 2ө = 23.0, 25.8, 27.7, 31.4 and 45.5 corresponded to hkl = 222, 026, 040, 044 and 408, respectively which were quite identical to the characteristic peaks of the S crystal.

### Disease assessment

**Fig. 5:**
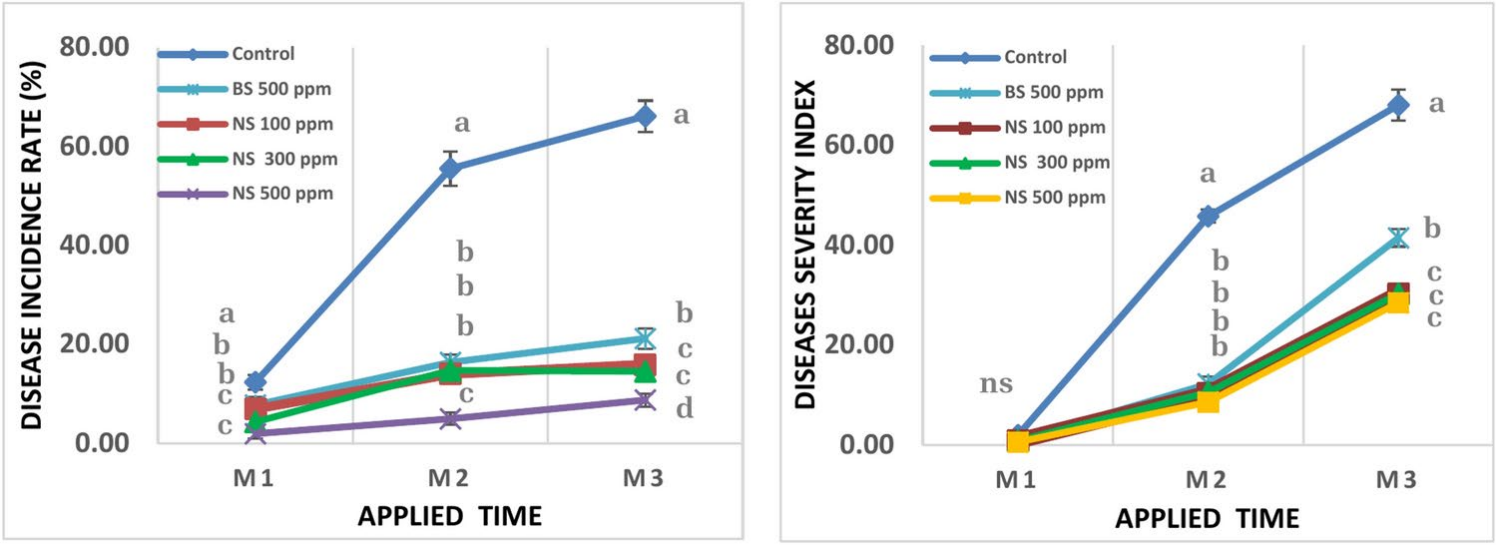
Effect of Sulfur nanoparticles foliar application on (**A**) Disease incidence rate% (**B**) Diseases severity index of powdery mildew in mango cv. Keitt. Values are means ± SE from five replicates (*n* = 5). Same letter means no significant differences between the values (*p* < 0.05) according to the Tukey test.

The foliar application of sulfur nanoparticles significantly inhibited the disease incidence rate and disease severity index of Powdery Mildew in mango cv. Keitt (Fig. 5). The inhibitory effect was more detectable after the second and third rounds of spraying, as treatment with 500 ppm nano sulfur had the lowest significant effect on the disease incidence rate, followed by treatment with 100 ppm and 300 ppm using nano sulfur, while there was no significant difference in the disease severity index. The highest disease incidence rate after the first, second, and third times of spraying (12.40, 55.53, and 66.14%, respectively) and disease severity index (2.00, 45.80, and 68.06, respectively) were obtained in the control treatment not sprayed with sulfur. while the treatment using bulk sulfur at 500 ppm gave average values between them for disease incidence rate after the first, second, and third times of spraying (7.80, 16.41, and 21.23%, respectively) and disease severity index (1.00, 12.28, and 41.49, respectively).

**Fig. 6:**
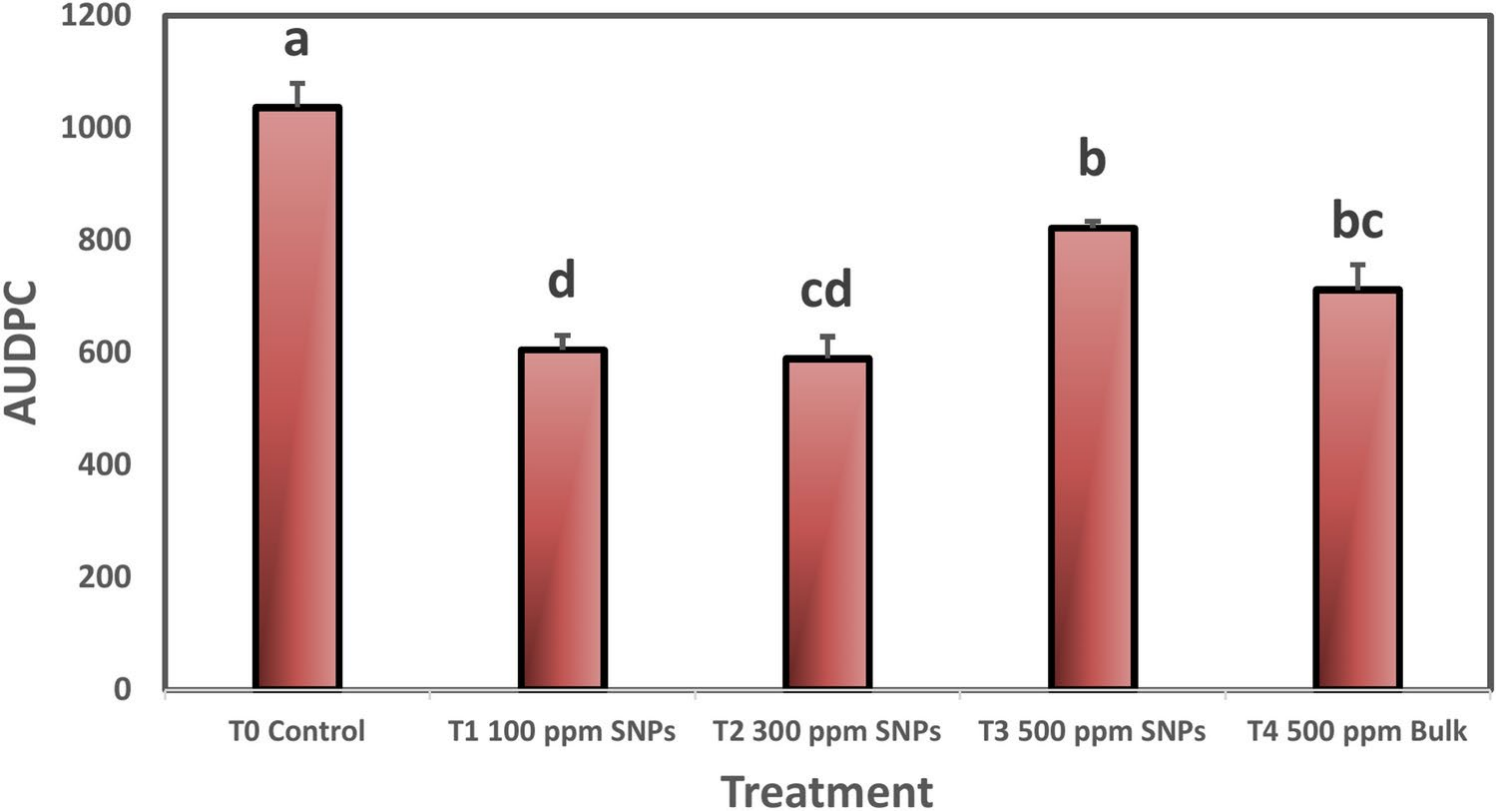
Effect of foliar spraying of nanocompounds on AUDPC for powdery mildew disease of mango cv. Keitt. Values are means ± SE from five replicates (n = 5). Same letter means no significant differences between the values (*p* < 0.05) according to the Tukey test.

Spraying sulfur nanoparticles caused a significant decrease in Area under disease progress curve (AUDPC) for all tested nanoparticles compared to the untreated control (Fig. 6). Using SNPs with 100 and 300 ppm showed the highest decrease in AUDPC, followed by 500 ppm bulk, while using SNPs with a concentration of 500 ppm showed the least significant decrease in the AUDPC.

**Fig. 7:**
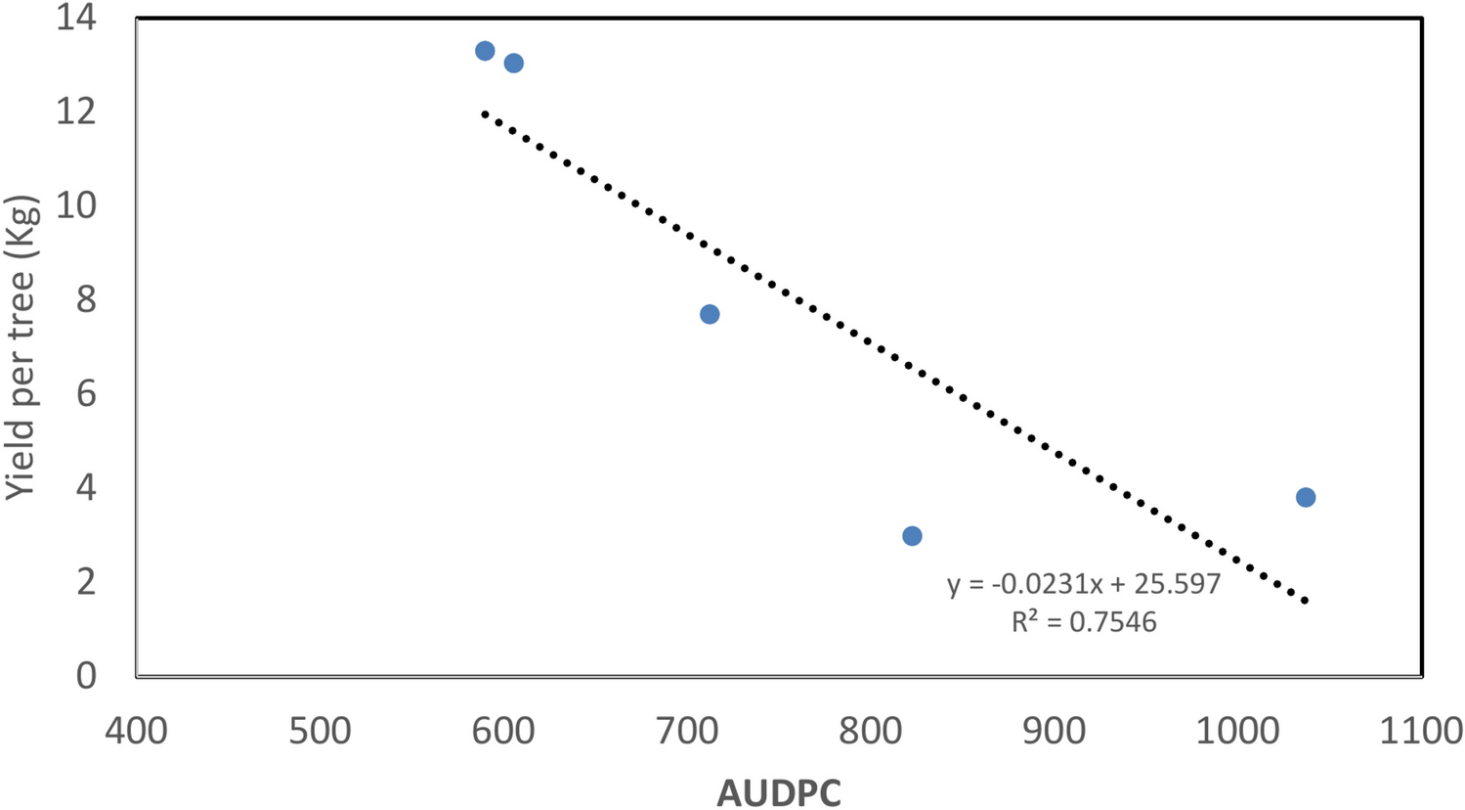
A strong negative correlation between AUDPC and average yield per tree due to powdery mildew infection, recording r =  − 0.87.

The correlation coefficient (r) between AUDPC and yield per tree due to the spraying with nanoparticles compared to control plants was analyzed as shown in Fig. 7. A strong negative correlation was observed between AUDPC recording and yield per tree, r =  − 0.87.

### Sulfur (S) content of leaves

**Fig. 8:**
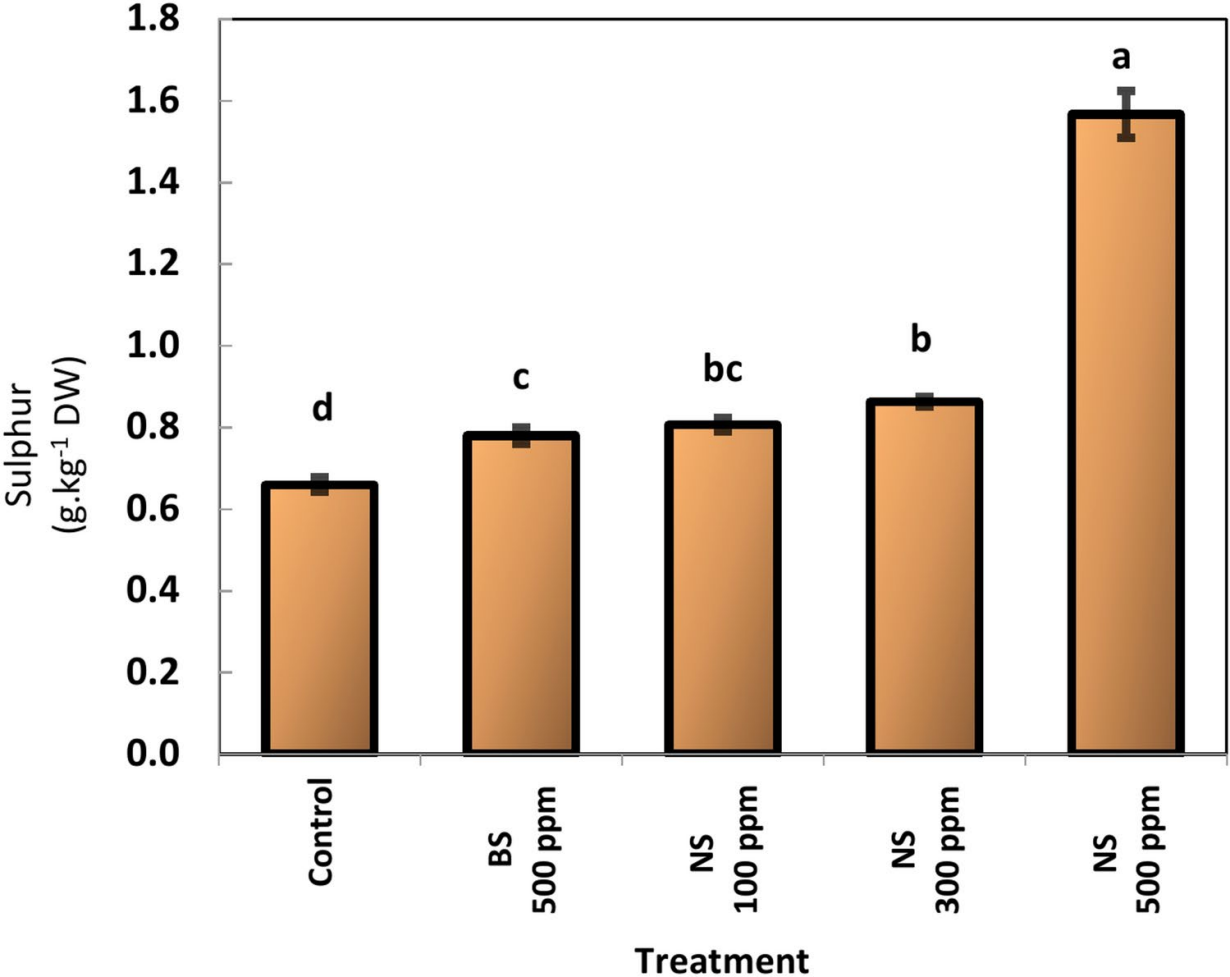
Effect of Sulfur nanoparticles foliar application on Sulphur (S) content of mango Cv. Keitt leaves. Values are means ± SE from five replicates (*n* = 5). Same letter means no significant differences between the values (*p* < 0.05) according to the Tukey test. ^*^Sulphur (S) content of mango leaves 0.37–0.88%^46^.

The sulfur content in the leaves of the mango cultivar Keitt increased with increasing treatment with sulfur, especially sulfur nanoparticles (Fig. 8). The highest sulfur contents were obtained with 500 ppm nano sulfur foliar application, for which the value was 1.57, which is greater than maximum sulfur content in mango leaves (0.37–0.88 according to^39^). On the other hand, the second highest sulfur content was obtained by 100 ppm nano sulfur, followed by nano sulfur 300 ppm foliar application (0.81 and 0.86 respectively), however, these values were within the optimal limits for the sulfur content of mango leaves.

### Tree productivity

**Fig. 9:**
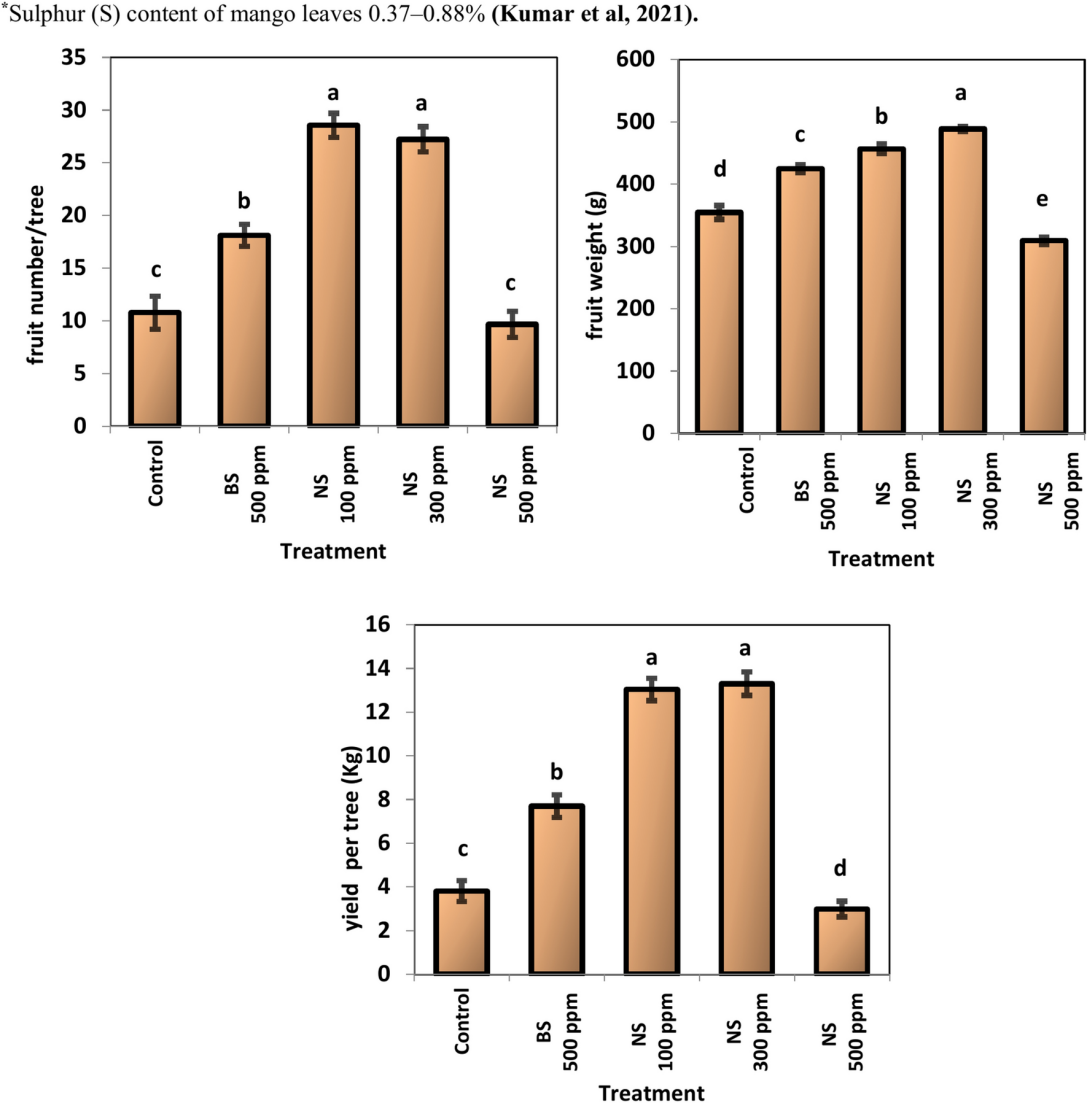
effect of Sulfur nanoparticles foliar application on productivity of mango Cv. Keitt . Values are means ± SE from five replicates (*n* = 5). Same letter means no significant differences between the values (*p* < 0.05) according to the Tukey test.

The productivity of Keitt mango trees under the experimental conditions as measured by fruit number per tree, the average weight of the fruit, and yield per tree were affected by the sulfur nanoparticle foliar application (Fig. 9). Nano sulfur 100 ppm and nano sulfur 300 ppm gave the highest significant values of fruit number (28.56 and 27.22, respectively) and yield per tree (13.04 and 13.30 kg). On the other hand, the lowest values of fruit number per tree, fruit weight and yield per tree were obtained by nano sulfur 500 ppm at a decreasing rate 33.85, 63.30, and 22.48%, respectively, compared to the highest values.

### Fruit quality

The physical and chemical characteristics of fruits, which are represented by fruit length, fruit diameter, fruit shape, pulp weight, peel thickness, TSS, acidity%, TSS/acid ratio, and vitamin C, were affected by experimental variables (Tables 1, 2 and 3). The fruits of mango trees showed the highest length and diameter which treated with bulk sulfur 500 ppm (124.81 and 84.18 mm, respectively), nano sulfur 100 ppm (125.44 and 86.39 mm, respectively), and nano sulfur 300 ppm (128.23 and 88.25 mm, respectively). Moreover, the fruit of control and nano sulfur 500 ppm treatments tend to be more elongated in shape, so they gave the highest fruit shape values by 1.53 (Table 1). Whereas the highest pulp weight of fruits was obtained by nano sulfur 300 ppm foliar application followed by nano sulfur 100 ppm foliar application by 175 and 162%, respectively, compared to nano sulfur 500 ppm foliar spraying. On the other hand, the lowest value of peel thickness was obtained by control treatment (68.68 mm) (Table 2).

**Table 1:** Effect of Sulfur nanoparticles foliar application on Length, diameter and shape of mango cv. Keitt fruit.

Treatment	Length (mm)	Diameter (mm)	Fruit shape
Control	113.70 ± 3.128^b^	74.58 ± 3.149^b^	1.53 ± 0.061^a^
Bulk sulfur 500 ppm	124.81 ± 2.946^a^	84.18 ± 2.815^a^	1.48 ± 0.058^a^
Nano sulfur 100 ppm	125.44 ± 3.154^a^	86.39 ± 3.895^a^	1.45 ± 0.065^ab^
Nano sulfur 300 ppm	128.23 ± 1.230^a^	88.25 ± 3.069^a^	1.45 ± 0.047^ab^
Nano sulfur 500 ppm	107.18 ± 3.140^c^	76.84 ± 2.221^b^	1.40 ± 0.057^b^

Means not sharing the letters for each variable in each column vary significantly at *p* ≤ 0.05 according to the Tukey test.

**Table 2:** Effect of Sulfur nanoparticles foliar application on Pulp weight and peel thickness of mango cv. Keitt fruit.

Treatment	Pulp weight (g)	Peel thickness (mm)
Control	307.00 ± 10.332^d^	68.68 ± 2.747^c^
Bulk sulfur 500 ppm	379.11 ± 6.679^c^	75.84 ± 2.757^a^
Nano sulfur 100 ppm	417.22 ± 4.353^b^	73.24 ± 3.873^ab^
Nano sulfur 300 ppm	451.33 ± 4.770^a^	73.84 ± 2.039^a^
Nano sulfur 500 ppm	257.22 ± 7.293^e^	69.38 ± 4.238^bc^

Means not sharing the letters for each variable in each column vary significantly at *p* ≤ 0.05 according to the Tukey test.

**Table 3:** Effect of Sulfur nanoparticles foliar application on TSS (Brix˚), Acidity, TSS/acid ratio and vitamin C of mango cv. Keitt fruit.

Treatment	TSS	Acidity%	TSS/acid ratio	Vit. C
Control	16.22 ± 0.507^bc^	2.28 ± 0.187^a^	7.16 ± 0.543^b^	30.48 ± 1.985^c^
Bulk sulfur 500 ppm	16.00 ± 0.935^c^	1.58 ± 0.164^b^	10.25 ± 1.346^a^	37.33 ± 2.390^b^
Nano sulfur 100 ppm	17.50 ± 0.354^a^	1.76 ± 0.172^b^	10.07 ± 1.193^a^	46.30 ± 3.170^a^
Nano sulfur 300 ppm	16.83 ± 0.250^ab^	1.66 ± 0.174^b^	10.23 ± 1.209^a^	40.07 ± 3.288^b^
Nano sulfur 500 ppm	16.00 ± 0.612^c^	1.54 ± 0.219^b^	10.54 ± 1.424^a^	19.02 ± 3.426^d^

Means not sharing the letters for each variable in each column vary significantly at *p* ≤ 0.05 according to the Tukey test.

Concerning chemical characteristics including TSS and Vitamin C. Nano sulfur 100 ppm foliar application gave the best results regarding the aforementioned parameters by 17.50 Brix and 46.30 mg/100 ml respectively, compared to the other treatments. In contrast, the lowest values of acidity% were shown in the fruits of trees treated with nano sulfur 100 ppm as a foliar application (Table 3).

**Fig. 10:**
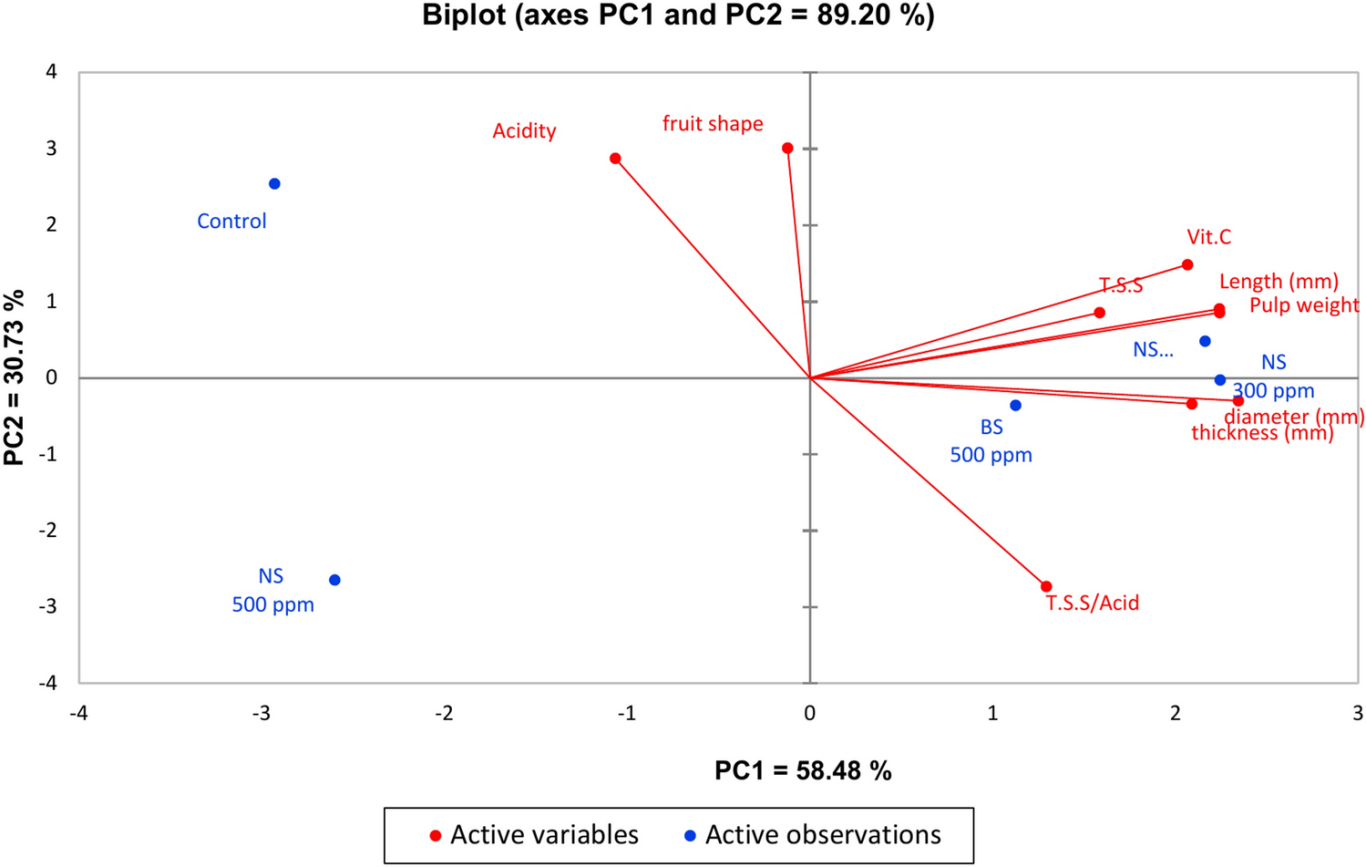
Principal component analysis (PCA) biplot for the assessed treatments of Sulfur nanoparticles foliar application and the physical and biochemical fruit quality attributes of mango Cv. Keitt.

Principal component analysis (PCA) was used to examine the relationship among the evaluated treatments of sulfur nanoparticles foliar application and the physical and biochemical fruit quality attributes of mango cv. Keitt, as shown in Fig. 10. The first two principal components accounted for 89.20% of the variability. The PCA1 represented 58.48% of the variation and was associated with the level of assessed treatments of sulfur nanoparticles foliar application from the untreated control on the extreme left to the highest level on the extreme right: nano sulfur 100 ppm and nano sulfur 300 ppm treatments. Nano sulfur 100 ppm and nano sulfur 300 ppm foliar applications had slight multidimensional space, as exhibited by the small distances of plots along PCA1, compared to the control and nano sulfur 500 ppm foliar applications which were spread apart and with more dissimilarity. Fruit length, pulp weight, TSS, TSS/acid ratio, and vitamin C were positively associated with nano sulfur 100 ppm and nano sulfur 300 ppm treatments in PCA1, which is consistent with the obtained results in Tables 1, 2, and 3. Thereupon, the PCA biplot reinforces the aforementioned results.

### Oxidative stress markers

**Fig. 11:**
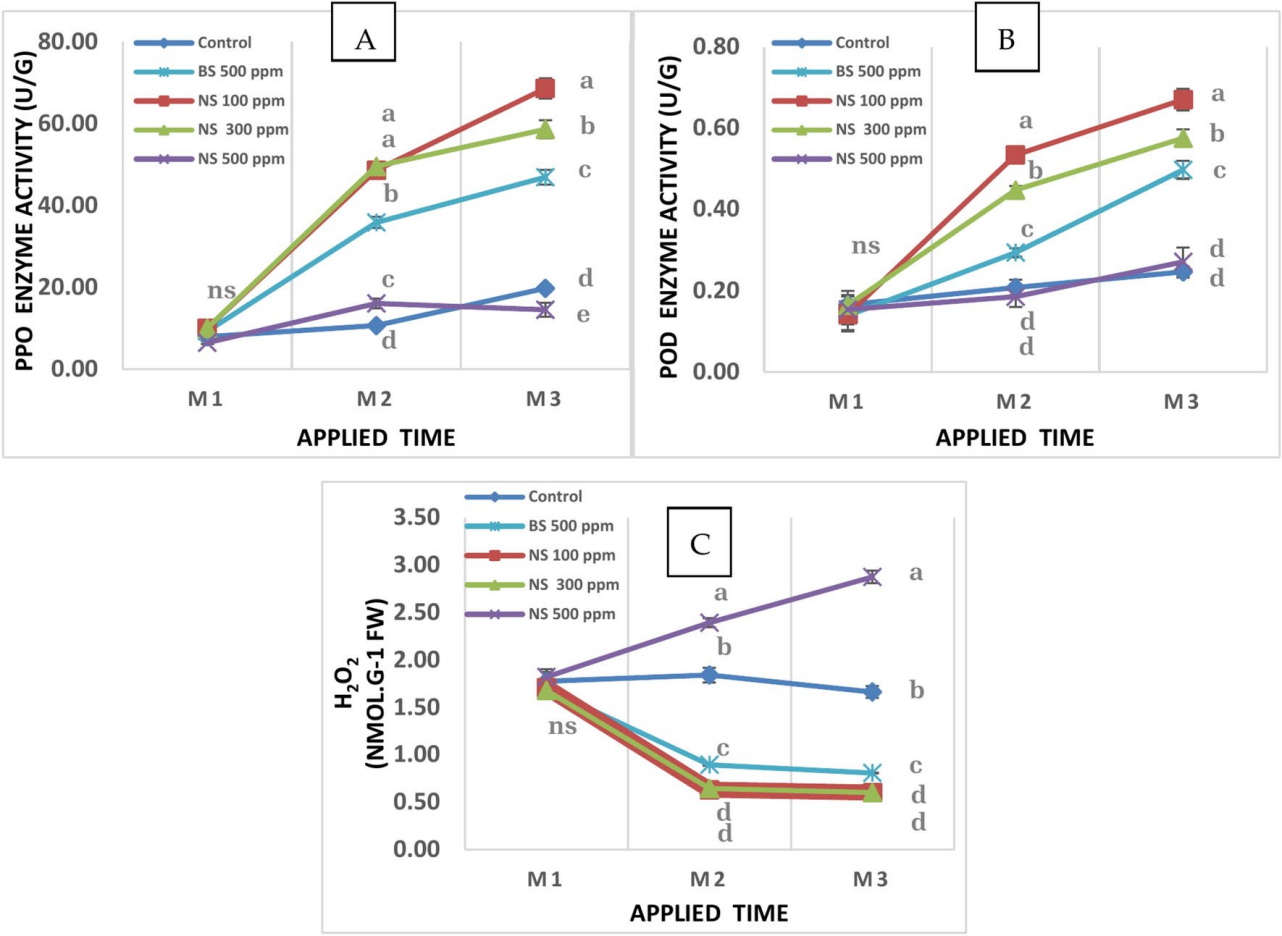
Effect of Sulfur nanoparticles foliar application on (**A**) PPO activity (**B**) POD activity (**C**) H_2_O_2_ of mango Cv. Keitt. Values are means ± SE from five replicates (*n* = 5). Same letter means no significant differences between the values (*p* ≤ 0.05) according to the Tukey test.

As expected, mango trees were infected with powdery Mildew disease, as a kind of biotic stress exhibited in an increase in H_2_O_2_ content. Generally, spraying sulfur at normal limits, except nano sulfur at a concentration of 500 ppm helped to increase PPO and POD activity which caused a reduction in the H_2_O_2_ content of plants especially after the second and the third application (Fig. 11A–C). The highest activity of PPO and POD enzymes was obtained with the 100 ppm nano sulfur by up to 472 and 248%, respectively; 300 ppm nano sulfur by up to 403 and 214%, respectively, compared to treated trees with bulk sulfur at 500 ppm. Spraying nano sulfur at a concentration of 100 ppm and 300 ppm helped to reduce the H_2_O_2_ content of plants followed by the treatment using bulk sulfur at 500 ppm (0.60, 0.60 and 0.81 nmol/g FW, respectively). While the highest values of H_2_O_2_ content showed in treatment by nano sulfur 500 ppm (2.88 nmol/g FW).

## Discussion

Powdery mildew is the most economically damaging disease in mango trees in Egypt and is caused by *Oidium mangiferae* Berthet fungus, which damages inflorescence, young leaves, and small fruits. Disease, as a biotic stress, interferes with the plant’s physiological functions including photosynthesis and respirations^22^, leading to loss of productivity more than 90% of tree yield^18,47^. This study aimed to alleviate the biotic stress caused by powdery mildew in mango cv. Keitt by using sulfur in a nanometric form, as sulfur application is well known as a preventive procedure in the powdery mildew control program^24,25^. Moreover, the use of nanoforms may increase the advantages of sulfur element.

### Disease assessment

Foliar application of sulfur obviously reduced the disease incidence rate and disease severity index of powdery mildew. It is worth mentioning that nano sulfur at concentrations of 100 ppm, 300 ppm and 500 ppm significantly surpassed bulk sulfur in defeating powdery mildew. Moreover, the superior performance of nanoform may be due to the presence of the ultrafine particles of nano sulfur, that chemically more reactive and bioactive than larger particles^28^. For the environmental aspects and to avoid the risk of phytotoxicity, sulfur nanoparticles could be used to best control of powdery mildew disease in low concentration 100 or 300 ppm as they didn’t differ significantly from 500 ppm treatment which achieved the lowest disease severity and incidence rate. The inhibitory effect of sulfur nanoform against powdery mildew may be by activating the salicylic acid-dependent systemic acquired resistance pathway like in tomato^31^. Furthermore, nanoparticles have safer and more cost-effective antibacterial and antioxidant effects than bulk forms^48^.

### Sulfur (S) content of leaves

NPs enhanced the increase in sulfur content in mango leaves because of their distinct physicochemical characteristics and superparamagnetic nature, which altered the rate of ion transport across the plasma membrane or otherwise impacted the structure of cell membrane lipid protein dynamics. This may have an impact on the permeability of the plasma membrane^49^. When the concentration of nano sulfur was 500 ppm gave values higher than optimal values of the sulfur content in the mango leaves was greater than the optimal values (0.37–0.88) according to ^46^, Furthermore, the sulfur content in the leaves may exceed the serious excessive limit (˃ 1.22 g/kg) according to^50^, which is phytotoxic to mango trees. A specific type and concentration of nanoparticle can occasionally have both advantageous and harmful impacts on the same plant^51^. The excessive use of sulfur nanoparticles has led to phytotoxicity. Although the exact mechanism underlying the phytotoxicity produced by SNPs is unknown, an imbalance in the antioxidant system may be the cause of this phenomenon. Overt leaf damage was also noticeable at apply 200 mg/L of SNPs foliar application on tomato^31^. Nanotoxicity mechanisms are still not fully understood, but are likely linked to the chemical composition, structure, size, and surface area of nanoparticles. The toxicity of nanoparticles is generally attributed to two main factors: (1) chemical toxicity due to the composition of the material, such as the release of toxic ions, and (2) stress stimuli from the surface, size, or shape of the particles^52^.

### Tree productivity

Powdery mildew attacks inflorescences, fruits, and leaves. The affected leaves become deformed, as a powdery coating on foliage and branches can lead to leaf loss and dieback. The affected fruits drop off prematurely and the yield is reduced. Sulfur treatments increased tree productivity by reducing the damage caused by powdery mildew, except for treatment with sulfur nanoparticles at a concentration of 500 ppm, which caused a decrease in productivity, perhaps due to toxicity, as the sulfur concentration in mango leaves exceeded the optimal limits. Additionally, most plant functions, including cellular structure, electron transport, and various metabolic pathways, have been shown to depend on sulfur. Since sulfur (S) is a vital component of proteins, amino acids, and a variety of secondary metabolites, it is necessary for the growth and development of plants^53^. Sulfur is thought to be safer than the other nanomaterials used in the agricultural industry because it can be incorporated into organosulfur compounds within plant tissues, which is necessary for the healthy growth of plants and strong antimicrobial activity^54^. The green synthesis of SNP at an optimal concentration of 1 mg/ml improved the physiological parameters in the lettuce plants increasing their tolerance of stressful conditions. However, higher concentration of SNP (10 mg/ml) indicated toxic effects on all the physiological parameters^55^. Nano sulfur addition resulted in the highest number of cucumber fruits per plant, as well as the highest mean weight of fruits per plant and mean weight of an individual fruit^29^.

### Fruit quality

Compared with those in the other treatments, the mango trees in the 100 and 300 ppm SNPs treatments were healthier, which was reflected in their productivity and fruit quality, as trees treated with 100 and 300 ppm SNPs treatments had better physical and chemical fruit quality characteristics. According to the literature, tomato plants treated with 200 ppm SNPS had greener and healthier leaves. The reason for these results is that SNPs received by leaves interact with organic compounds found in tomato tissues to generate organic sulfur compounds. These compounds aid in the development of leaf chlorophyll and nitrogen contents. It contributed to the plants’ increased organic component content, producing tomato fruits of high quality in comparison to the control group. Additionally, tomato leaves treated with 200 ppm SNPs were found to be healthier and greener than control leaves. This suggests that the right concentration of SNPs is needed for the organic components in plant tissue to produce organic-sulfur compounds, which aid in the healthy growth, self-defense, and antibacterial action of plants^35,36^.

### Oxidative stress markers

Overproduction of reactive oxygen species (ROS; hydrogen peroxide H_2_O_2_, superoxide O_2_^−^; hydroxyl radical OH^−^ and singlet oxygen ^1^O_2_) is enhanced under abiotic and/or biotic stresses, which can result in oxidative damage to plant macromolecules and cell structures, ultimately hindering plant growth and development, or even causing death^56^. Biotic stress caused by powdery mildew infection increases the hydrogen peroxide (H_2_O_2_) content in plants. SNPs at concentration of 100 ppm and 300 ppm inhibited the increase in H_2_O_2_ output from powdery mildew infection in this study, indicating that SNPs at concentration of 100 ppm and 300 ppm lessened the induction of oxidative stress by increasing PPO and POD enzyme activity. At 500 ppm, SNPs decreased PPO and POD enzyme activity and increased H_2_O_2_, and the excessive use of SNPs at 500 ppm led to phytotoxicity. An imbalance in the antioxidant system may be the cause of this difference. After pretreatment with 100 µM SNPs, the non-enzymatic antioxidant contents of the plants improved. In addition, SNPs therapy reversed the excessively high levels of catalase (CAT), peroxidase (POD), ascorbate peroxidase (APX), superoxide dismutase (SOD), and polyphenol oxidase (PPO)^54^. Several studies have demonstrated that plants exhibit increased activities of PPO and POD that correlated with an increased diseases resistance^57,58^. Furthermore, sulfur plays a key role in antioxidant activity and photosynthesis. It is also linked to resistance against abiotic and biotic stress, as well as secondary metabolism^59^. In addition^60^, noted that sulfur in the form of nanoparticles displayed a potent anti-nematicidal activity against *Meloidogyne javanica* invasion. Enzymatic antioxidants and other defense mechanisms, such as sulfur-containing substances such as the vital macronutrient sulfur (S), glutathione, a class of phytochelatins, S-rich proteins, S-amino acids, hydrogen sulfide (H_2_S), and a variety of secondary metabolites, are involved in the stress response^61^. Emerging research suggests that sulfur nanoparticles could play a role in mitigating oxidative stress. These nanoparticles possess antioxidant properties that can scavenge ROS and reduce their harmful effects. By neutralizing ROS, sulfur nanoparticles may help alleviate oxidative stress and contribute to overall cellular health. Studies have shown that the introduction of sulfur nanoparticles into biological systems can enhance the activity of antioxidant enzymes such as SOD and CAT. Moreover, sulfur nanoparticles can modulate the expression of genes involved in oxidative stress pathways, leading to a reduction in oxidative damage.

## Conclusions

The present study aimed to investigate the effect of sulfur nanoparticles (SNPs) on enhancing the yield and productivity of Keitt mango cultivar by decreasing the incidence rate and severity index of powdery mildew disease. The study found that SNPs foliar application significantly reduced disease incidence rate and severity index. The most obvious effect was observed after the second and third sprayings. Additionally, SNPs foliar application increased the sulfur content of mango leaves, improved tree productivity, and affected the physical and chemical characteristics of fruits. The study also found that spraying SNPs helped to reduce the H_2_O_2_ content of plants, especially after the second and third application. Sulfur nanoparticles possess antioxidant properties that can scavenge ROS and reduce their harmful effects. Accordingly, spraying Keitt cultivar with SNPs at 100 ppm is regarded as an advisable practice to get better yield and productivity of mango exposed to powdery mildew disease. Although the highest level of SNPs showed higher resistance against disease but exhibited the lowest performance of trees and productivity. By neutralizing ROS, sulfur nanoparticles may help alleviate oxidative stress and contribute to overall cellular health. However, excessive use of sulfur nanoparticles can lead to phytotoxicity.

## Supplementary data

All the data generated or analyzed during this study are included in this published article.

## Data Availability

Data is provided within the manuscript.

## References

[1] Galán Saúco, V. Trends in world mango production and marketing. *Acta Horticult.* **1183**, 351–364. 10.17660/actahortic.2017.1183.51 (2017).

[2] Welay, P., Gebreyesus, B. T., Mulugeta, B. & Johan, M. Effectiveness of water-saving techniques on growth performance of mango (*Mangifera indica* L.) seedlings in Mihitsab-Azmati Watershed, Rama Area. Northern Ethiopia. *Agric Water Manag* **243**, 106476. 10.1016/j.agwat.2020.106476 (2021).

[3] Abdel Samad, A. G. A. & Shaaban, A. Fulvic and salicylic acids improve morpho-physio-biochemical attributes, yield and fruit quality of two mango cultivars exposed to dual salinity and heat stress conditions. *J Soil Sci Plant Nutr* **24**, 6305–6324. 10.1007/s42729-024-01968-7 (2024).

[4] MALR (Ministry of Agriculture and Land Reclamation). Bulletin of agricultural statistics, Arab republic of Egypt. The Egyptian Economic Affairs Sector. Dokki, Egypt. https://www.agri.gov.eg/uploads/-topics/17329628961779.pdf (2023).

[5] Muhammed, M. A., Mohamed, A. K. S., Qayyum, M. F., Haider, G. & Ali, H. A. Physiological response of mango transplants to phytohormones under salinity stress. *Sci. Hortic.* **296**, 110918. 10.1016/j.scienta.2022.110918 (2022).

[6] Kumar, R. & Kumar, V. Physiological disorders in perennial woody tropical and subtropical fruit crops-A review. *Indian J. Agric. Sci.* **86**, 703–717 (2016).

[7] Kulkarni, S., Chavan, T. & Vijaykumar, K. N. Management of mango powdery mildew through potential organic products. *IJBSM* **14**(5), 756–761. 10.23910/1.2023.3360 (2023).

[8] Reuveni, M., Gur, L. & Farber, A. Development of improved disease management for powdery mildew on mango trees in Israel. *Crop. Prot.* **110**, 221–228. 10.1016/j.cropro.2017.07.017 (2018).

[9] Ratnadass, A. & Deguine, J. P. Three-way interactions between crop plants, phytopathogenic fungi, and mirid bugs. A review. *Agron. Sustain. Dev.* **40**, 46. 10.1007/s13593-020-00652-1 (2020).

[10] Nofal, M. A. & Haggag, W. M. Integrated management of powdery mildew of mango in Egypt. *Crop Prot.* **25**, 480–486. 10.1016/j.cropro.2005.08.003 (2006).

[11] Braun, U. & Cook, R. T. A. Taxonomic manual of the Erysiphales (powdery mildews). *CBS Biodivers. Ser.* **11**, 497 (2012).

[12] Deguine, J. P. et al. Agroecological protection of mango orchards in La Réunion. In *Sustainable Agriculture Reviews* Vol. 28 (eds Gaba, S. et al.) 249–307 (Springer, Cham, 2018). 10.1007/978-3-319-90309-5_8.

[13] El-Meslamany, R. A., Atia, M. M. & Abd-Elkader, D. A. Evaluation of cultivars and fungicides role in controlling mango powdery mildew. *Plant Prot. Pathol. Res.* **47**, 87–100. 10.21608/zjar.2020.70124 (2020).

[14] Vielba-Fernández, A. et al. Fungicide resistance in powdery mildew fungi. *Microorganisms* **8**, 1–34. 10.3390/microorganisms8091431 (2020). 10.3390/microorganisms8091431 PMC7564317 32957583

[15] Mapuranga, J. et al. Infection strategies and pathogenicity of biotrophic plant fungal pathogens. *Front. Microbiol.* **13**, 799396. 10.3389/fmicb.2022.799396 (2022). 35722337 10.3389/fmicb.2022.799396 PMC9201565

[16] Nasir, M. et al. Powdery mildew of mango: a review of ecology, biology, epidemiology and management. *Crop Prot.* **64**, 19–26. 10.1016/j.cropro.2014.06.003 (2014).

[17] Shukla, P. K., Adak, T. T. & Gundappa, G. Seasonal dynamics of powdery mildew of mango and its management under subtropics. *GERF Bull Biosci* **7**(1), 21–25 (2016).

[18] Pérez-Rodríguez, A., Monteón-Ojeda, A., Mora-Aguilera, J. A. & Hernández-Castro, E. Epidemiology and strategies for chemical management of powdery mildew in mango. *Pesqui. Agropecu. Bras.* **52**, 715–723. 10.1590/S0100-204X2017000900003 (2017).

[19] Khaskheli, M. I. Mango diseases: Impact of fungicides. In *Horticultural Crops* (eds Kossi, B. H., Hamamouch, N., Adjiguita, K. Y.) (IntechOpen, 2020). 10.5772/intechopen.87081

[20] Deresa, E. M. & Diriba, T. F. Phytochemicals as alternative fungicides for controlling plant diseases: A comprehensive review of their efficacy, commercial representatives, advantages, challenges for adoption, and possible solutions. *Heliyon* **9**, e13810. 10.1016/j.heliyon.2023.e13810 (2023). 36879959 10.1016/j.heliyon.2023.e13810 PMC9984788

[21] Arora, A. et al. Management of powdery mildew (*Oidium mangiferae*) of mango with fungicides. *Plant Dis. Res.* **36**, 58–61. 10.5958/2249-8788.2021.00009.3 (2021).

[22] Williams, J. S. & Cooper, R. M. The oldest fungicide and newest phytoalexin—a reappraisal of the fungitoxicity of elemental sulphur. *Plant Pathol.* **53**, 263–279. 10.1111/j.0032-0862.2004.01010.x (2004).

[23] Gupta, R. C. & Crissman, J. W. Chapter 42—Agricultural chemicals. In *Haschek and Rousseaux’s Handbook of Toxicologic Pathology* 3rd edn, Vol. 2 (eds Haschek, W. M., Rousseaux, C. G., Wallig, M. A.) 1349–1372 (Academic Press**, **2013). 10.1016/B978-0-12-415759-0.00042-X.

[24] Amich, J. Sulfur metabolism as a promising source of new antifungal targets. *J. Fungi* **8**, 295. 10.3390/jof8030295 (2022). 10.3390/jof8030295 PMC8951744 35330297

[25] Wu, P. H., Chang, H. X. & Shen, Y. M. Effects of synthetic and environmentally friendly fungicides on powdery mildew management and the phyllosphere microbiome of cucumber. *PLoS One* **18**, 1–16. 10.1371/journal.pone.0282809 (2023). 10.1371/journal.pone.0282809 PMC9994715 36888572

[26] Wang, T. et al. Elemental sulfur inhibits yeast growth via producing toxic sulfide and causing disulfide stress. *Antioxidants* **11**, 576. 10.3390/antiox11030576 (2022). 35326226 10.3390/antiox11030576 PMC8945482

[27] Abou Seeda, M. A. et al. Importance of sulfur and its roles in plants physiology: A review. *Curr. Sci. Int.* **9**, 198–231. 10.36632/csi/2020.9.2.18 (2020).

[28] Gogoi, R. et al. Suitability of nano-sulphur for biorational Management of Powdery mildew of Okra (*Abelmoschus esculentus Moench*) caused by *Erysiphe cichoracearum*. *J. Plant Pathol. Microbiol.* **4**, 1–4. 10.4172/2157-7471.1000171 (2013).

[29] Abdel-Rahman, H. & El-Kollaly, A. Comparative studies on nanosulfur and certain fungicides to control cucumber powdery mildew disease and their residues in treated fruits. *Alex. Sci. Exch. J.* **41**, 53–60. 10.21608/asejaiqjsae.2020.74243 (2020).

[30] Wang, Y., Deng, C., Sharma, S., et al. Nanoscale-specific bioassimilation of sulfur: Time and coating specific modulation of transcriptomic and metabolomic pathways in diseased tomato. 09, PREPRINT (Version 1) available at Research Square 10.21203/rs.3.rs-1128848/v1 (2021)

[31] Cao, X. et al. Elemental sulfur nanoparticles enhance disease resistance in tomatoes. *ACS Nano* **15**, 11817–11827. 10.1021/acsnano.1c02917 (2021). 34148346 10.1021/acsnano.1c02917

[32] Abou El-Nasr, M. K. et al. Using zinc oxide nanoparticles to improve the color and berry quality of table grapes Cv. Crimson seedless. *Plants* **10**, 1285. 10.3390/plants10071285 (2021). 34202840 10.3390/plants10071285 PMC8309036

[33] Turganbay, S. et al. Synthesis and characterization of sulfur nanoparticles with WSP/surfactants mixtures. *Int. J. Biol. Chem.* **12**, 146–52. 10.26577/ijbch-2019-1-i19 (2019).

[34] Yuan, H. et al. Sulfur nanoparticles improved plant growth and reduced mercury toxicity via mitigating the oxidative stress in *Brassica napus* L. *J. Clean. Prod.* **318**, 128589. 10.1016/j.jclepro.2021.128589 (2021).

[35] Salem, N. M., Al-banna, L. S. & Abdeen, A. O. Sulfur nanoparticles improves root and shoot growth of tomato. *J. Agric. Sci.* **8**, 179–185. 10.5539/jas.v8n4p179 (2016).

[36] Salem, N. M., Albanna, L. S. & Awwad, A. M. Green synthesis of sulfur nanoparticles using *Punica granatum* peels and the effects on the growth of tomato by foliar spray applications. *Environ. Nanotechnol. Monit. Manag.* **6**, 83–87. 10.1016/j.enmm.2016.06.006 (2016).

[37] Shiv, S. et al. Preparation of sulfur nanoparticles and their antibacterial activity and cytotoxic effect. *Mater. Sci. Eng. C* **92**, 508–517. 10.1016/j.msec.2018.07.015 (2018). 10.1016/j.msec.2018.07.015 30184776

[38] Campbell, C. L. & Madden, L. V. *Introduction to Plant Disease Epidemiology* (Wiley, New York, 1990).

[39] Gülüt, K. Y. & Hosgökdelen, B. Sulfur and nitrogen nutrition status in flag leaf and shoot samples collected from wheat growing areas in Çukurova, Central Anatolia and GAP regions of Turkey. *Saudi J. Biol. Sci.* **28**, 4807–4817. 10.1016/j.sjbs.2021.05.010 (2021). 34354470 10.1016/j.sjbs.2021.05.010 PMC8325053

[40] AOAC. *Official Method and Analysis, Association of the Official Analytical Chemists* 16th edn (Washington DC, 1995).

[41] Nielsen, S. S. Vitamin C determination by indophenol method. In *Food Analysis Laboratory Manual. Food Science Text Series* (eds Nielsen, S. S.) (Springer, Cham, 2017). 10.1007/978-3-319-44127-6_15

[42] Oktay, M. et al. Polyphenol oxidase from Amasya apple. *J. Food Sci.* **60**(3), 494–496. 10.1111/j.1365-2621.1995.tb09810.x (1995).

[43] Polle, A., Otter, T. & Seifert, F. Apoplastic peroxidases and lignification in needles of Norway Spruce (*Picea abies* L.). *Plant Physiol.* **106**, 53–60. 10.1104/pp.106.1.53 (1994). 12232302 10.1104/pp.106.1.53 PMC159498

[44] Velikova, V., Yordanov, I. & Edreva, A. Oxidative stress and some antioxidant systems in acid rain-treated bean plants: Protective role of exogenous polyamines. *Plant Sci.* **151**, 59–66. 10.1016/S0168-9452(99)00197-1 (2000).

[45] Komorowski, M., Marshall, D. C., Salciccioli, J. D. & Crutain, Y. Exploratory data analysis. In *Secondary Analysis of Electronic Health Records* 185–203 (Springer, Cham, 2016). 10.1007/978-3-319-43742-2_15 31314267

[46] Kumar, M. et al. Mango (*Mangifera indica* L.) leaves: Nutritional composition, phytochemical profile, and health-promoting bioactivities. *Antioxidants* **10**, 1–23. 10.3390/antiox10020299 (2021). 10.3390/antiox10020299 PMC7920260 33669341

[47] Misra, A. K. Powdery mildew-A serious disease of mango. *J. Appl. Hortic.* **3**, 63–68. 10.37855/jah.2001.v03i01.16 (2001).

[48] Shahbaz, M. et al. Evaluation of selenium nanoparticles in inducing disease resistance against spot blotch disease and promoting growth in wheat under biotic stress. *Plants* **12**, 1–22. 10.3390/plants12040761 (2023). 10.3390/plants12040761 PMC9958785 36840109

[49] Stange, B. C., Rowland, R. E., Rapley, B. I. & Podd, J. V. ELF magnetic fields increase amino acid uptake into *Vicia faba* L. roots and alter ion movement across the plasma membrane. *Bioelectromagnetics* **23**, 347–354. 10.1002/bem.10026 (2002). 12111755 10.1002/bem.10026

[50] Huang, C. et al. Nutritional diagnosis of the mineral elements in Tainong mango leaves during flowering in Karst areas. *Land* **11**, 1311. 10.3390/land11081311 (2022).

[51] Pacheco, I.& Buzea, C. Nanoparticle uptake by plants: Beneficial or detrimental? In: *Phytotoxicity of Nanoparticles* 1–61 (Springer International Publishing, 2018). 10.1007/978-3-319-76708-6

[52] Ruttkay-Nedecky, B. et al. Nanoparticles based on essential metals and their phytotoxicity. *J. Nanobiotechnol.* **15**, 1–19. 10.1186/s12951-017-0268-3 (2017). 10.1186/s12951-017-0268-3 PMC5406882 28446250

[53] Suleiman, M. et al. Synthesis of nano-sized sulfur nanoparticles and their antibacterial activities. *J. Mater. Environ. Sci.* **6**, 513–518 (2015).

[54] Saad-Allah, K. M. & Ragab, G. A. Sulfur nanoparticles mediated improvement of salt tolerance in wheat relates to decreasing oxidative stress and regulating metabolic activity. *Physiol. Mol. Biol. Plants* **26**, 2209–2223. 10.1007/s12298-020-00899-8 (2020). 33268924 10.1007/s12298-020-00899-8 PMC7688864

[55] Najafi, S., Razavi, S. M., Khoshkam, M. & Asadi, A. Effects of green synthesis of sulfur nanoparticles from *Cinnamomum zeylanicum* barks on physiological and biochemical factors of Lettuce (*Lactuca sativa*). *Physiol. Mol. Biol. Plants* **26**, 1055–1066. 10.1007/s12298-020-00793-3 (2020). 32377053 10.1007/s12298-020-00793-3 PMC7196604

[56] Hossain, M. A. et al. Hydrogen peroxide priming modulates abiotic oxidative stress tolerance: insights from ROS detoxification and scavenging. *Front. Plant Sci.* **6**, 1–19. 10.3389/fpls.2015.00420 (2015). 26136756 10.3389/fpls.2015.00420 PMC4468828

[57] Ji, Y. et al. Effect of Ethanol Vapor treatment on the growth of *Alternaria alternata* and *Botrytis cinerea* and defense-related enzymes of fungi-inoculated blueberry during storage. *Front. Microbiol.* **12**, 618252. 10.3389/fmicb.2021.618252 (2021). 33574808 10.3389/fmicb.2021.618252 PMC7870470

[58] Feng, L., Sun, J., Jiang, Y. & Duan, X. Role of reactive oxygen species against pathogens in relation to postharvest disease of papaya fruit. *Horticulturae* **8**(3), 205. 10.3390/horticulturae8030205 (2022).

[59] Capaldi, F. R. et al. Sulfur metabolism and stress defense responses in plants. *Trop. Plant Biol.* **8**, 60–73. 10.1007/s12042-015-9152-1 (2015).

[60] Al Banna, L. S., Salem, N. M., Jaleel, G. A. & Awwad, A. M. Green synthesis of sulfur nanoparticles using Rosmarinus officinalis leaves extracts and anti-nematicidal activity against *Meloidogyne javanica*. *Chem. Int.* **6**, 137–143. 10.5281/zenodo.3528019 (2020).

[61] Gratão, P. L. et al. Biochemical dissection of *diageotropica* and *Never ripe* tomato mutants to Cd-stressful conditions. *Plant Physiol. Biochem.* **56**, 79–96. 10.1016/j.plaphy.2012.04.009 (2012). 22609458 10.1016/j.plaphy.2012.04.009

